# Supplementary material for: Development and Validation of a New Screening Tool with Non-Invasive Indicators for Assessment of Malnutrition Risk in Hospitalised Children
Source: Children (Basel). 2022 May 17;9(5):731. doi: 10.3390/children9050731 (PMC9140013; doi:10.3390/children9050731)
Supplement: Supplementary file 1 [file children-09-00731-s001.zip › children-1690856-SM.pdf]

Supplementary Material S1.

Inclusion and exclusion criteria.

**Inclusion criteria**	
Research sample/population:	Hospitalized children from one month to 18 years old with expected hospital stay (LOS) > 24 hours, regardless of the cause of admission or the child's medical condition.<br>Parents and/or child able to speak Slovenian.
**Exclusion criteria**	
Infants during the first month of life.<br>Expected hospital stay (LOS) < 24 hours.<br>Outpatient patients.<br>Patients in intensive care units (ICU).<br>Hospitalized children in whom anthropometric measurements could not be performed.<br>Parents and / or children who did not agree to participate in this research<br>Patients with COVID-19.	

Supplementary Material S2.

Questionnaire´s content sets.

**Content set**	**Description**
Basic patient and admission data	Age, gender, unit of admission.
Diagnosis and treatments	Admission diagnosis, presents of chronical diseases, state of chronical disease, allergies, diets, gastrointestinal malformation, cachexia, gastrointestinal operative interventions, antibiotic therapy, continuing medicine therapy.
Anthropometric measurements	Weight (past, present), height, skin folds, body circumferences, weight loss.
Body examination	Loss of subcutaneous fat, loss of muscle mass, presents of physical signs (exp. visible ribs, thin neck, alopecia, …).
Oedema/ascites	The presence of oedema throughout the body, the presence of ascites.
Vital functions	Blood pressure, pulse, SpO2, respiration rate, heigh metabolic needs.
Dietary intake	Feeding, presence of oral-motor dysfunctions /disorders, current and past dietary intake, refusal of food, symptoms and diseases affecting food intake, extremes in restricting feeding, problems with chewing or swallowing of food, behavioural eating problems, improper eating habits.

Content set	Description
Gastrointestinal symptoms	Nausea, vomiting, constipation, diarrhea, the presents of abdominal pain, changed appetite, taste or smell, presents of mucositis or sore throat.
Psychological factors	consumption of non-food products, low self-esteem.
Stress factors	The presents of different stress factors, strict parenting upbringing.
Body temperature	Measure.
Resistance and tissue regeneration	Presence of infections, wounds, or bleeding.
Physical endurance	Signs of exhaustion, disorientation, dizziness, physical weakness, poor physical performance, irritability, extreme physical exercise.
Social/emotional functions	Unsociability, mental numbness, depression, apathy, insomnia.
Menstrual cycle	Presents and regularity of menstruation.

Supplementary Material S3.

Indicators of malnutritional status using anthropometric measures [58].

Kazalnik	Mild Malnutrition	Moderate Malnutrition	Severe Malnutrition
Weight-for-height *z* score	−1 to −1.9 *z* score	−2 to −2.9 *z* score	−3 or greater *z* score
BMI-for-age *z* score	−1 to −1.9 *z* score	−2 to −2.9 *z* score	−3 or greater *z* score
Length/height-for-age z score	No data	No data	−3 *z* score
Mid–upper arm circumference	Greater than or equal to −1 to −1.9 *z* score	Greater than or equal to −2 to −2.9 *z* score	Greater than or equal to −3 *z* score

BMI : body mass index

Supplementary Material S4.

Characterization of the study sample.

	Involved in the study (n = 180)	Development phase (n=142)	Validation phase (n=38)
Male:Female n (%)	86 (47.8):94 (52.2)	69 (48.6):73 (51.4)	17 (44.7):21 (55.3)
Age in months (Median (95% CI))	130 (108, 142)	123 (93, 138)	143 (113, 169)
**Reason of admission**	**n (%)**	**n (%)**	**n (%)**
Nephrology and arterial hypertension	93 (51.7)	77 (54.2)	16 (42.1)
Gastroenterology, hepatology, and nutrition	42 (23.3)	31 (21.8)	11 (28.9)
General paediatrics	16 (8.9)	12 (8.5)	4 (10.5)

	Involved in the study (n = 180)	Development phase (n=142)	Validation phase (n=38)
Neurology	12 (6.7)	10 (7.0)	2 (5.3)
Pulmonology, rheumatology and allergology	10 (5.6)	6 (4.2)	4 (10.5)
Pedo-psychiatry	7 (3.9)	6 (4.2)	1 (2.6)
**Prevalence of malnutrition**	**n (%)**	**n (%)**	**n (%)**
**Subjective malnutritional risk assessment [a]**			
Severe risk of malnutrition	10 (5.6)	7 (4.9)	3 (7.9)
Moderate risk of malnutrition	31 (17.2)	25 (17.6)	6 (15.8)
Mild risk of malnutrition	28 (15.6)	22 (15.5)	6 (15.8)
Normal or healthy body weight	79 (43.9)	63 (44.4)	16 (42.1)
Risk of overweight	13 (7.2)	10 (7.0)	3 (7.9)
Risk of obesity	19 (10.6)	15 (10.6)	4 (10.5)
**WFH [b]**	**n (%)**	**n (%)**	**n (%)**
Severe malnutrition	2 (1.1)	2 (1.4)	0 (0.0)
Moderate malnutrition	10 (5.6)	8 (5.6)	2 (5.3)
Mild malnutrition	22 (12.2)	17 (12.0)	5 (13.2)
Absence of malnutrition	146 (81.1)	115 (81.0)	31 (81.6)
**BMI [b, c]**	**n (%)**	**n (%)**	**n (%)**
Severe malnutrition	7 (3.9)	4 (2.8)	3 (7.9)
Moderate malnutrition	18 (10.0)	17 (12.0)	1 (2.6)
Mild malnutrition	21 (11.7)	14 (9.9)	7 (18.4)
Absence of malnutrition	134 (74.5)	107 (75.4)	27 (71.1)
**HFA [b]**	**n (%)**	**n (%)**	**n (%)**
Severe malnutrition	3 (1.7)	3 (2.1)	0 (0.0)
Moderate malnutrition	3 (1.7)	3 (2.1)	0 (0.0)
Mild malnutrition	18 (10.0)	13 (9.2)	5 (13.2)
Absence of malnutrition	156 (86.6)	123 (86.6)	33 (86.8)
**MUAC [b]**	**n (%)**	**n (%)**	**n (%)**
Severe malnutrition	5 (2.8)	3 (2.1)	2 (5.3)
Moderate malnutrition	15 (8.3)	11 (7.7)	4 (10.5)
Mild malnutrition	37 (20.6)	29 (20.4)	8 (21.1)
Absence of malnutrition	123 (68.3)	99 (69.7)	24 (63.2)
**Assessment of malnutrition when at least one of the above indicators is present [b]**	**n (%)**	**n (%)**	**n (%)**
Severe malnutrition	8 (4.4)	5 (3.5)	3 (7.9)
Moderate malnutrition	23 (12.8)	19 (13.4)	4 (10.5)
Mild malnutrition	41 (22.8)	33 (23.2)	8 (21.1)
Absence of malnutrition	108 (60.0)	85 (59.9)	23 (60.6)
**Overweight and obesity according to international standards [d]**	**n (%)**	**n (%)**	**n (%)**
Overweight	8 (4.4)	5 (3.5)	3 (7.9)
Obesity	23 (12.8)	19 (13.4)	4 (10.5)

[a] : by physician, [b] : defined by Becker et al. and as recommended by the ASPEN and Academy of Nutrition and Dietetics [58]; [c] : by WHO; [d] : according to international standards [59]; WFH: weight-for-height/length, BMI: Body mass index, HFA: Height-for-age, MUAC: Mid upper arm circumference.
